# Genomic Instability and Radiation Risk in Molecular Pathways to Colon Cancer

**DOI:** 10.1371/journal.pone.0111024

**Published:** 2014-10-30

**Authors:** Jan Christian Kaiser, Reinhard Meckbach, Peter Jacob

**Affiliations:** Institute of Radiation Protection, Helmholtz Zentrum München, Deutsches Forschungszentrum für Gesundheit und Umwelt (GmbH), Neuherberg, Germany; University of Hawaii Cancer Center, United States of America

## Abstract

Colon cancer is caused by multiple genomic alterations which lead to genomic instability (GI). GI appears in molecular pathways of microsatellite instability (MSI) and chromosomal instability (CIN) with clinically observed case shares of about 15–20% and 80–85%. Radiation enhances the colon cancer risk by inducing GI, but little is known about different outcomes for MSI and CIN. Computer-based modelling can facilitate the understanding of the phenomena named above. Comprehensive biological models, which combine the two main molecular pathways to colon cancer, are fitted to incidence data of Japanese a-bomb survivors. The preferred model is selected according to statistical criteria and biological plausibility. Imprints of cell-based processes in the succession from adenoma to carcinoma are identified by the model from age dependences and secular trends of the incidence data. Model parameters show remarkable compliance with mutation rates and growth rates for adenoma, which has been reported over the last fifteen years. Model results suggest that CIN begins during fission of intestinal crypts. Chromosomal aberrations are generated at a markedly elevated rate which favors the accelerated growth of premalignant adenoma. Possibly driven by a trend of Westernization in the Japanese diet, incidence rates for the CIN pathway increased notably in subsequent birth cohorts, whereas rates pertaining to MSI remained constant. An imbalance between number of CIN and MSI cases began to emerge in the 1980s, whereas in previous decades the number of cases was almost equal. The CIN pathway exhibits a strong radio-sensitivity, probably more intensive in men. Among young birth cohorts of both sexes the excess absolute radiation risk related to CIN is larger by an order of magnitude compared to the MSI-related risk. Observance of pathway-specific risks improves the determination of the probability of causation for radiation-induced colon cancer in individual patients, if their exposure histories are known.

## Introduction

Cancer is caused by multiple genomic alterations which lead to genomic instability. Two principal molecular forms of genomic instability have been observed in tissue from colorectal tumors. High-level microsatellite instability (MSI) appears if DNA mismatch repair (MMR) genes are defective. MSI tumors exhibit frequent mutations in short repeated DNA sequences called micro-satellites. About 15–20% of sporadic cases are related to MSI which often starts with silencing of the MMR gene *MLH1* by promoter methylation. Chromosomal instability (CIN) constitutes the second form of genomic instability which is less clearly defined. CIN tumors show a large heterogeneity in chromosomal copy number and structure (named aneuploidy), whereas MSI tumors are near-diploid with few karyotypic abnormalities. CIN is associated with the loss of wild-type copies of tumor suppressor genes (TSGs) such as *APC*, *TP53* or *SMAD4* which regulate the growth and death of cells with tumorigenic mutations. CIN tumors are micro-satellite stable through effective mismatch repair. About 80–85% of colorectal tumors are of the CIN (or low-level MSI) type. However, CIN and MSI can share molecular properties such as mutations in the *BRAF* gene or the CpG island methylator phenotype (CIMP). The pathways are not mutually exclusive and a more refined pathway classification has been suggested [1]–[8].

About 20–30% of patients with colorectal cancer possess a familial risk with two or more first or second degree relatives having colorectal cancer but only 5–10% of all patients develop the disease in a strictly inherited manner [9]. The two main phenotypes are hereditary nonpolyposis colorectal cancer (HNPCC or Lynch syndrome) and familial adenomatous polyposis (FAP) [10]. FAP constitutes the hereditary form of the CIN pathway. A germline mutation in the APC gene has been detected in about 80% of FAP patients [9]. FAP and attenuated FAP are also related to bi-allelic inherited mutations of the *MutYH* gene without showing *APC* mutations [11]. HNPCC is associated with the MSI pathway and causes about 3% of colorectal tumors [10].

Environmental factors and lifestyle can also influence the formation of colorectal cancer but the impact may vary in colon and rectum [12]. For the present analysis, which is focused on the life span study (LSS) of a-bomb survivors, the Japanese setting is of special interest. A westernized diet has been identified as an important cause for the increase of colon cancer incidence in Japan [13]. Incidence rates converged to those in the US population from the late 1950s to the mid-1990s [14].

A number of mathematical models have been developed to represent the most important biological processes of colon carcinogenesis. A model with a single path to colon cancer has been fitted to incidence data of the Surveillance, Epidemiology and End Results (SEER) registry [15]. *APC* loss was included as the promoter of genetic chaos consistent with Knudson's two-hit paradigm of oncogenesis. At the same time a conceptual model has been proposed which considered CIN as an early event [16]. Cells carrying a CIN property together with silenced *APC* genes are expected to show markedly elevated mutation rates compared to cells with silenced *APC* genes alone. Both models were included in a comparative analysis of five mechanistic models and a descriptive model which have been fitted to the SEER data [17]. Based on goodness-of-fit criteria no clear winner emerged from this exercise. An explorative study of models which explicitly aimed to identify traits of MSI and CIN in the SEER data did not produce compelling evidence [18]. Typical time scales for the development of precancerous lesions and tumor growth in different sites of the gastro-intestinal tract (including the colon) have been detected by applying multi-stage clonal expansion (MSCE) models to SEER data [19], [20]. MSI tumors are rarely found in the distal colon [2]. Compared to the proximal colon slightly faster adenoma growth has been observed in the distal colon, possibly caused by differential oncogenic dynamics of the CIN and MSI pathways [21]. None of the precursor models unambiguously discovered imprints of distinct molecular pathways for incidence data of colorectal cancer in the SEER cohort.

Mechanisms that relate radiation to genomic instability are still not fully explained [22]–[24]. Radiation-induced genomic instability and other molecular radiation effects have been mimicked in biologically-based models of carcinogenesis for several organs [25]. To estimate radiation risks simple two-stage models of initiation and promotion have been applied to incidence data from the LSS cohort [26], [27]. These mechanistic models with radiation effects relied on a uniform description of tumorigenic processes which did not address organ-specific peculiarities.

For many organs, including the colon, estimates of the excess absolute risk (EAR) and excess relative risk (ERR) are derived from the LSS data with descriptive models [28]. Such estimates are considered as the accepted standard by committees BEIRVII [29], ICRP [30] and UNSCEAR [31], which issue recommendations for radiation protection. They are applied in compensation claims from nuclear workers and US army veterans [32]. Particularly for colon cancer, reliable risk coefficients are needed for a risk-benefit analysis of mass screening by computer tomography colonography (CTC) [33].

In the present study biologically-based modelling is applied to detect imprints of pertinent tumorigenic processes for colon cancer in the LSS incidence data. It is aimed to reproduce the share of clinically observed cases in the MSI and CIN pathways. For both sexes the total risk and pathway-specific risks are compared to standard risk coefficients from descriptive models.

## Materials and Methods

### LSS dataset of colon cancer incidence

In August 1945, residents of Hiroshima and Nagasaki were acutely exposed to a mixed field of γ-radiation and neutrons from two a-bomb explosions. Individual radiation doses are represented in the latest dosimetry system DS02 [34]. For the neutron contribution to the total colon dose a weight of ten is used which is motivated by higher biological effectiveness. Incidence data for solid cancers were collected from 1956 onwards for 120 321 members of the LSS cohort to assess late health effects. Subjects came from all age groups and were not selected for pre-existing illness.

The LSS cohort was created as a stratified random sample of the entire available population of such survivors, including all of the available survivors who had been exposed to the bombs at proximal distances. In addition to extensive collection of demographic and exposure data in the early years after cohort inception, the cohort has been followed for mortality using nationwide data in Japan and for cancer incidence by tumor registries established in both Hiroshima and Nagasaki. Operation of the Hiroshima and Nagasaki tumor registries is reviewed regularly by the institutional review boards of the Radiation Effects Research Foundation (RERF) and the registries. Protocols used for vital status and cause of death ascertainment in the LSS are regularly reviewed by the RERF board. The protocols include the assurance that patient information would be kept confidential and grant permission to access the stored information. With the approval of the tumor registries, the RERF cohorts are routinely linked with the registries to identify tumors among cohort members. The full LSS data set is publicly available in file *lssinc07.csv* from the RERF website. The set consists of 24 205 completely anonymised Poisson records in grouped form which prevents the identification of individual patient information.

Although carcinogenesis acts very similar in colon (ICD10:C18) and rectum (ICD10:C20), the rectum data are discarded in the present study. The radiation risk for the rectum is negligible in the LSS [28]. Person years (PY) and cases in excess of 4 Gy shielded air kerma have been excluded to avoid modelling of deterministic radiation effects. These exclusions reduce the number of colon cancer cases by 8 (5 male/3 female) to 1508 (Table A4 in ref. [28]). A summary of the LSS data for colon cancer incidence is given in Table 1.

**Table 1:** Summary of colon cancer incidence data in the LSS cohort from 1958–1998 for dose groups with shielded air kerma <4 Gy, 95% percentiles of frequency distribution for person years in brackets.

	Men	Women	Both sexes
Subjects	42 762	62 384	105 146
Person Years (PY)×10^6^	1.04	1.72	2.76
Cases	688	820	1508
PY-weighted mean age at exposure *e* (yr)	21 (1; 52)	24 (1; 52)	23 (1; 52)
Case-weighted mean age at exposure *e* (yr)	25 (1; 52)	30 (2; 54)	28 (2; 53)
PY-weighted mean attained age *a* (yr)	50 (17; 80)	55 (19; 83)	53 (18; 82)
Case-weighted mean attained age *a* (yr)	67 (43; 86)	71 (45; 87)	69 (44; 87)
PY-weighted mean colon dose *D* (Gy)	0.083 (0; 0.75)	0.079 (0; 0.66)	0.081 (0; 0.69)
Case-weighted mean dose *D* (Gy)	0.142 (0, 1.2)	0.092 (0; 0.84)	0.115 (0;1.1)

The person-year weighted mean dose of about 0.081 Gy for both sexes combined is very similar to the subject-weighted mean dose of 0.083 Gy (0.085 Gy male, 0.081 Gy female). The case-weighted mean dose for both sexes combined is 0.12 Gy. A higher value for the case-weighted mean indicates an association of colon cancer and radiation. But the association appears notably weaker in women than in men. To confirm this observation, relative risks of groups with low to moderate (0.005–0.25 Gy) dose and moderate to high (>0.25 Gy) doses have been calculated compared to the unexposed (<0.005 Gy) population. The crude data show a significant relative risk in the group with moderate to high doses only for men or for both sexes combined (Table 2). Relative risks from crude data are only indicative and cannot replace a proper risk assessment study.

**Table 2:** Relative risk (RR) with 95% CI in colon dose groups 0.005–0.25 Gy and>0.25 Gy compared to dose group <0.005 Gy.

Dose group	RR Men	RR Women	RR Both sexes
0.005–0.25 Gy	1.05 (0.89; 1.24)	0.99 (0.85; 1.14)	1.01 (0.90; 1.13)
>0.25 Gy	1.77 (1.42; 2.21)	1.03 (0.81; 1.31)	1.35 (1.14; 1.58)

### Mechanistic model

The present cell-based model for the two main molecular pathways to colon cancer (in short two path (TP) model) relies on the concept of growth control for precancerous lesions by caretaker and gatekeeper genes [35], [36]. Although cell alignment and spatial movement play a role in tumorigenesis [37], the two path model is only concerned with the kinetics of mutations and cell growth.

**Figure 1:**
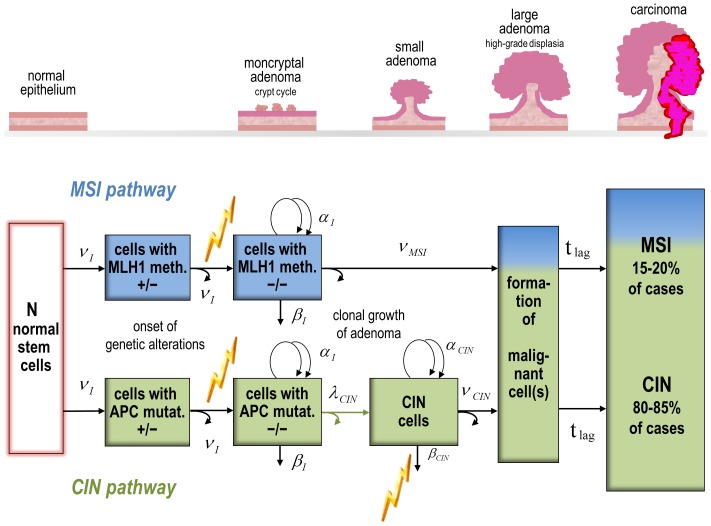
Conceptual model for colon cancer carcinogenesis from normal epithelium to carcinoma with two molecular pathways of genomic instability: microsatellite instability (MSI, top, blue) and chromosomal instability (CIN, bottom, green). Greek symbols denote rates of mutation or hypermethylation (ν) as genetic alterations successively on both alleles, and rates of symmetric cell division (α) or inactivation (β); genetically altered cells are created by asymmetric cell division (marked by a pair of straight and bent arrows, for normal stem cells only the straight arrow is used to account for homeostasis); the rate λ_CIN_ of destabilizing events in CIN (pair of green arrows) depends on birth cohort; in large adenoma at least one malignant cell leads to a tumor, which is detected after a fixed lag time t_lag_ = 5 yr; jagged bolts (yellow) point to radiation targets of the preferred two path model TP4.

Colon epithelium consists of a single cell layer organized in finger-shaped crypts. Each of the many million crypts houses a small stem cell population in a niche at the bottom. The total population of healthy stem cells is in homeostasis and can reproduce all intestinal cell types by asymmetric division [37]. This process generally creates one altered daughter cell and leaves the other daughter cell unchanged. In the simplified conceptual model of Figure 1 the pathways to cancer are initiated either by bi-allelic mutation of the *APC* gene (CIN) or by bi-allelic methylation of the *MLH1* gene (MSI) [1]. Both genetic alterations are generated in asymmetric cell division. The baseline rates ν_I1_ and ν_I2_ for genetic alterations in the first and second hit cannot be determined independently [19]. These two successive rates have been set equal but differences between pathways were allowed. No further assumptions for the cell kinetics in the two path model were made.

Clonal expansion of cells with tumorigenic mutations generates neoplastic lesions, which undergo further transitions on the way to cancer. Clonal growth of initiated cells is a stochastic process, in the early stage clones may die out or survive. Growth of adenoma starts with surviving clones in separate crypts (monocryptal adenoma). It is assumed that a cycle of crypt fission and extinction dominates clonal expansion in of premalignant cells at this early stage [38]. Crypt fission is a very slow process which occurs on average once in 2–3 decades [37]. In the two path model initiated cells either divide symmetrically with rate α_I_ or are inactivated (i.e. by apoptosis or extinction) with rate β_I_. A direct functional relation between the net growth rate γ_I_≈α_I_-β_I_ and the rate of crypt fission is not obvious, since γ_I_ belongs to events for single cells and crypt fission involves many cells. However, both rates depend on the same underlying cell kinetics and similar numerical values for such rates seem plausible. As an effective net parameter γ_I_ describes the growth dynamics together in monocryptal adenoma and in the crypt cycle equally for both pathways. Crypt fission at a normal rate is the mechanism which spreads inactivated TSGs such as *APC* or *MLH1* in the human colon [39]. During growth of early adenoma the transient patterns of MSI and CIN diverge possibly due to different effects of silenced TSGs in both pathways. In the model the pathways are treated as independent so that incidence rates for different pathways can be added to obtain the total incidence. However, in reality some molecular processes such as deregulated WNT signalling are found in both pathways [4]–[6], [8].

A single transforming mutation ν_MSI_ concludes the MSI path by creation of at least one malignant cell which leads to a tumor. Although the MSI path exhibits a higher degree of complexity, a simplification is justified by the small number of expected cancer cases from MSI [2].

The CIN pathway continues with a destabilizing event of rate λ_CIN_ which precedes clonal growth in larger adenoma. To account for lifestyle trends, λ_CIN_ is scaled by an exponential factor exp[l_b_(1915.6-*b*)] which increases with birth year *b*. The net rate of stochastic clonal growth γ_CIN_≈α_CIN_-β_CIN_ for CIN cells is determined by the difference between symmetric cell division α_CIN_ and inactivation β_CIN_. Transformation of CIN cells with mutation rate ν_CIN_ to at least one malignant cell, which leads to a tumor, is considered as the final rare event of tumorigenesis in the CIN pathway. In both pathways a fixed lag time t_lag_ = 5 yr is chosen for the duration until the first malignant cell grows into a clinically relevant tumor.

Radiation action has been assumed to increase the rate ν_I2_ of the second hit in initial mutations or in hypermethylation. Reduction of the inactivation rate β_CIN_ for CIN cells was applied as a second radiation effect (Figure 1). Reduced cell inactivation is a plausible mechanism to promote clonal growth [40]. Combined radiation action on cell division and inactivation or on division alone might also be considered but different radiation effects in promotion have negligible influence on the fit results. Radiation action on the destabilizing CIN event and other radiation targets (results not reported) have been tested as well. The statistical quality of model fits has been measured by the Akaike Information Criterion (AIC  =  deviance +2×no. of model parameters N_par_ [41]).

### Numerical solution of the two path model

The two path model fits into the mathematical framework of Little and Wright [42] who have generalized the two-step clonal expansion (TSCE) model introduced by Moolgavkar and Knudson [43]. The TSCE model relies on two rate-limiting mutations which are separated by clonal expansion of initiated cells. Mutation rates and rates of cell division or inactivation are treated as transient Poisson point processes of cell birth and death which are expressed in a set of master equations [44]. The approach to solve the TSCE model for piecewise constant model parameters has been extended to the larger set of master equations for the two path model [45]. This set has been transformed into a system of coupled differential equation of the Ricatti type which is solved efficiently by an approximate iterative algorithm for calculating the survival function. The hazard is obtained by numerical differentiation of the survival function. The total hazard of the two path model is given by the sum of the hazard for the separate MSI and CIN models. Mathematical derivations of equivalent models have been given in ref. [19] (MSI without t_lag_) and ref. [20] (CIN without t_lag_) in a notation which is applied in the present study in a similar way.

### Identification of model parameters

Eight different parameters for biological transition rates are shown in Figure 1. These rates should be at least in principle accessible for experimental investigation. But the differential equations for the two path model are couched in terms of less intuitive identifiable parameters. The identifiability problem follows from the mathematical model structure and cannot be removed by increasing statistical power [46]. In the so-called deterministic versions of the MSI and CIN models fluctuations in clone size are neglected. Since rates of TSG (*APC*, *MLH1*) inactivation and of early clonal expansion have been set equal after a series of statistical tests (see below), the four deterministic baseline parameters R_MSI_, γ_I_, R_CIN_ and γ_CIN_ can be identified in a fit. R_MSI_ and R_CIN_ pertain to the hazard of a simple Armitage-Doll model with multiplied mutations rates. In the present study the full stochastic versions of both models are used. They depend additionally on the two stochastic parameters δ_I_ and δ_CIN_ which account for fluctuations in clone size. During clone birth such fluctuations are important since they may lead to extinction. Relations between identifiable baseline parameters and biological transition rates are shown in Table S1 in File S1. Deterministic parameters often possess smaller uncertainties than stochastic parameters. Separation of stochastic effects from deterministic effects stabilizes the fitting procedure.

### Parameter estimation and uncertainty analysis

The MECAN software package has been used for pre-processing of the grouped data, regression, comparison of observed and expected cases, and simulation of uncertainty intervals [47]. The package is written in the C++ programming language. Its object-oriented design is based on separate libraries for processing of epidemiological data sets and for the introduction of new mechanistic or descriptive risk models. The libraries are linked to the computational core which performs the standard tasks of likelihood minimisation and simulation of uncertainties for risk estimates. Thanks to a high degree of standardisation, new projects of radio-epidemiological analysis can be set up with little programming effort. Parallelisation has been achieved by linking the code to functions of the OpenMP library (www.openmp.org).

MECAN includes the C++ library Minuit2 from CERN which is used for the minimization of −2 ln*L* where *L* denotes the Poisson likelihood [48]. The Poisson deviance is given by the minimum of −2 ln*L* which is reached with the maximum likelihood estimates (MLE) of the model parameters. It is assumed that a parabolic approximation of the region around the minimum is valid. In this case Wald-based standard errors (SE), confidence intervals (CI_LP_) from the actual likelihood profile and a correlation matrix can be computed for the model parameters. Confidence intervals (CI) for risk estimates are calculated by Monte-Carlo simulation. Results of MECAN were found to be in good agreement with the EPICURE package which is a standard software for the analysis of radio-epidemiological data [49].

For the conceptual model of Figure 1 with different identifiable baseline parameters for both pathways and both sexes. But different parameters have been kept in the model only if the fit was improved with a probability of at least 95% (or the deviance was lowered by at least 3.8 points) in a likelihood ratio test (LRT). Radiation dependent parameters have been added only if they passed the same LRT. More details on the statistical analysis approach of model parameter selection are given in ref. [50].

## Results

### Goodness-of-fit

In Table 3 Poisson deviance and AIC for the tested models are shown. In the mechanistic TP models the fixed lag time t_lag_ = 5 yr was counted as an additional model parameter, the remaining identifiable parameters have been determined by a fit. Two path model

**Table 3:** Deviance and ΔAIC values for the descriptive and mechanistic models of the present study, ΔAIC is defined as the difference in AIC to the preferred two path model TP4.

Symbol	Model specification	Radiation response parameters	Deviance	N_par_	ΔAIC
DERR^a^	Descriptive ERR	Sex-specific ERR	4485.5	20	29.4
DEAR^a^	Descriptive EAR	Sex-specific EAR	4489.4	20	33.3
M1^b^	Two initial mutations, promotion and malignant transformation	Sex-specific, lifelong on promotion	4495.5	7	13.4
M2^c^	Early genomic instability	Sex-specific on 2^nd^ destabilizing mutation	4476.0	14	7.9
M3^d^	Deterministic MSI path and stochastic CIN path, no early clonal expansion in CIN	Unisex on 2^nd^ hit of initiation ν_I_, for men only on CIN promotion γ_CIN_ (like TP4)	4479.0	10	2.9
TP0	Two path	No radiation effect	4507.9	9	29.8
TP1	Two path	Unisex on 2^nd^ hit of initiation ν_I_	4481.9	10	5.8
TP2	Two path	Unisex on 2^nd^ hit of initiation ν_I_, unisex on CIN promotion γ_CIN_	4477.1	11	3.0
TP3	Two path	Unisex on 2^nd^ hit of initiation, for men only on destabilizing CIN event λ_CIN_	4476.0	11	1.9
TP4^e^	Two path	Unisex on 2^nd^ hit of initiation ν_I_, for men only on CIN promotion γ_CIN_ (like M3)	4474.1	11	0.0

a from Preston et al. [28].

b baseline model equivalent to the three-stage model of Meza et al. [19], see SI, Table S2 and Figure S1 in File S1.

c inspired by Nowak et al. [16], derived from Little and Li [17] (their Figure 2), see SI, Table S3 and Figure S2 in File S1.

d Table S4 and Figure S3 in File S1.

e preferred model of the present study.

TP0 without a radiation effect provides the benchmark for models TP1 to TP4, which show similar goodness-of-fit for different radiation targets. Replacing radiation action for men only in model TP3 by unisex radiation action on the destabilizing CIN event did not improve the fit compared to model TP1. Model TP4 yielded the lowest Poisson deviance and AIC, and is preferred for risk assessment in the present study. MLE, SE and σCI_LP_ from the likelihood profile are given in Table 4 for the identifiable parameters. Radiation-dependent versions of the mechanistic models by Meza et al. [19] (M1, Figure S1 in File S1), and by Little and Li [17] (M2, Figure S2 in File S1) yielded AIC values which came out higher by 13 points and 8 points, respectively. Fitting a radiation-dependent version of the full MSCE model by Luebeck et al. [20] was not successful. Parameter estimates for models M1 and M2 are shown in Tables S2 and S3 in File S1. The baseline parameters of model M1 roughly agree with those of the three-stage model in ref. [19]. For model M2 a low deviance was achieved, but parameter estimates are notably different for both sexes. For men acceleration in subsequent phases of clonal growth was found but the opposite trend for women is biologically implausible. In model M3 the MSI and CIN pathways are treated jointly as in the TP models, but the first phase of clonal expansion has been omitted for the CIN path. Radiation acts similar in models M3 and TP4 (see Table S4 and Figure S3 in File S1). The MSI path of model M3 could be described deterministically since the effect of fluctuations in clone size was negligible (i.e. δ_MSI_ = α_MSI_ ν_T,MSI_≈0). Compared to model TP4 model M3 yielded a slightly inferior ΔAIC of 2.9 points. Preston et al. [28] developed descriptive models of the ERR (termed DERR) and EAR (termed DEAR) which were refitted to the present slightly restricted LSS dataset. Since the difference in results is negligible the reader is referred back to ref. [28] for an extensive discussion. AIC values of the descriptive models are about 30 points higher compared to the preferred two path model TP4.

**Table 4:** MLE, SE from a parabolic approximation around the minimum of the likelihood function, and ΔCI_LP_ from the actual likelihood profile in the standard σ range for the identifiable parameters of the two path model TP4 with relation to biological parameters, superscript m,f indicates sex-dependence, radiation-response parameters r_I_ and r^m^_CIN_ on dose *D* are given for an exposure duration of 1 week, rate λ_CIN_(b) of destabilizing events in CIN increases exponentially with birth year *b* = 1945.6–*e* (age at exposure).

Symbol	Unit	MLE	SE	ΔCI_LP_	Relation to biological parameters
R_MSI,0_	yr^−3^	−17.56^a^	0.33	−0,31; 0.30	= Nν_I_^2^ ν_MSI_ r(0)
r_I_	week Gy^−1^	6.89^a^	0.33	−0.38; 0.30	r(*D*) = 1+r_I_ *D*/week
γ_I_	yr^−1^	0.0568	0.013	−0.012; 0,012	= α_I_–β_I_-ν_MSI_
δ_I_	yr^−2^	−11.1^a^	1.1	−1.0; 0.9	= α_I_ ν_MSI_
R_CIN,0_	yr^−4^	−26.6^a^	2.0	not calc.	= Nν_I_^2^ λ_CIN_(1915.6) ν_CIN_ r(0)
l_b_	yr^−1^	−0.109	0.023	−0.021; 0.015	λ_CIN_(*b*) = λ_CIN_(1915.6) exp[l_b_ (1915.6-*b*)]
γ^f^_CIN,0_	yr^−1^	0.234	0.043	−0.022; 0.026	= α_CIN_ – β^f^_CIN_ – ν_CIN_
γ^m^_CIN,0_	yr^−1^	0.266	0.040	−0.020; 0.026	= α_CIN_ – β^m^_CIN_ g(0) – ν_CIN_
r^m^_CIN_	week Gy^−1^	5.68^a^	0.34	−0.40; 0.30	g(*D*) = 1-r^m^_CIN_ *D*/week
δ_CIN_	yr^−2^	−16.9^a^	2.4	−2.0; 1.4	= α_CIN_ ν_CIN_
t_lag_	yr	5 (fixed)			

a log-transformed.

### Biological parameters for cell-based processes

Applying LRTs on a 95% level for the removal of statistically insignificant parameters allowed to reduce model complexity. The initiating rates of the first and second hit were set equal ν_I1_ = ν_I2_ = ν_I_ since a pathway-specific treatment was rejected by appropriate LRTs. The rates of early clonal growth γ_I_ also came out very similar in both pathways and they have been set equal as well. Fitting two path models for both sexes separately produced similar mutation rates (including birth cohort dependences) and rates of early clonal growth. A distinction between sexes was not necessary for these parameters on the basis of LRTs. However, the relatively small difference for the sex-specific rates of clonal growth γ_CIN_ in late adenoma was highly significant. The deviance was increased by more than hundred points if the growth rates were set equal for both sexes.

From the estimates of identifiable parameters (Table 4) the biological baseline rates of the two path model TP4 can be derived, if assumptions on the total number of susceptible stem cells N, and the rates of symmetric cell division for initiated cells α_I_ and for destabilized CIN cells α_CIN_ are made. The number of stem cells has been estimated to approx. 10^8^ with an accuracy of an order of magnitude [51], [52]. MSI tumors appear mainly in the proximal colon so that a (by a factor of 2–3) lower number of susceptible stem cells might be considered for the MSI path [2]. However, the biological parameter N alone is not identifiable and the uncertainties in the estimates for parameters including N are too large to prove effects of different values in the LSS data. Thus, the same value for N has been applied in the MSI and CIN pathways to derive the inactivation rate ν_I_. Cell division rates of 9 yr^−1^ in adenoma and 29 yr^−1^ in early carcinoma have been reported [53]. If these values are assigned to α_I_ and α_CIN_, rates for the transforming mutations ν_MSI_, ν_CIN_, and the cell inactivation rates β_I_, β_CIN_ can be calculated. Values for biological baseline parameters, which describe the cell kinetics of the preferred two path model TP4, are summarized in Table 5.

**Table 5:** Estimates for baseline rates (unit yr^−1^ per cell) of cell-kinetic processes in the two path model TP4.

Cell-kinetic process	Symbol	Value
*APC* mutation or *MLH1* hypermethylation (1^st^ and 2^nd^ hit)	ν_I_	1.2×10^−5^
Cell inactivation in early adenoma (crypt cycle)	β_I_	8.943
Transforming mutation in MSI	ν_MSI_	1.7×10^−6^
Rate of destabilizing events in CIN (chromosomal gain or loss) for birth years *b* = 1895, 1915, 1935	λ_CIN_(*b*)	0.016, 0.14, 1.2
Cell inactivation in adenoma with CIN cells (male)	β^m^_CIN_	28.734
Cell inactivation in adenoma with CIN cells (female)	β^f^_CIN_	28.766
Transforming mutation in CIN	ν_CIN_	1.5×10^−9^

### Case shares and radiation risks in molecular pathways

**Figure 2:**
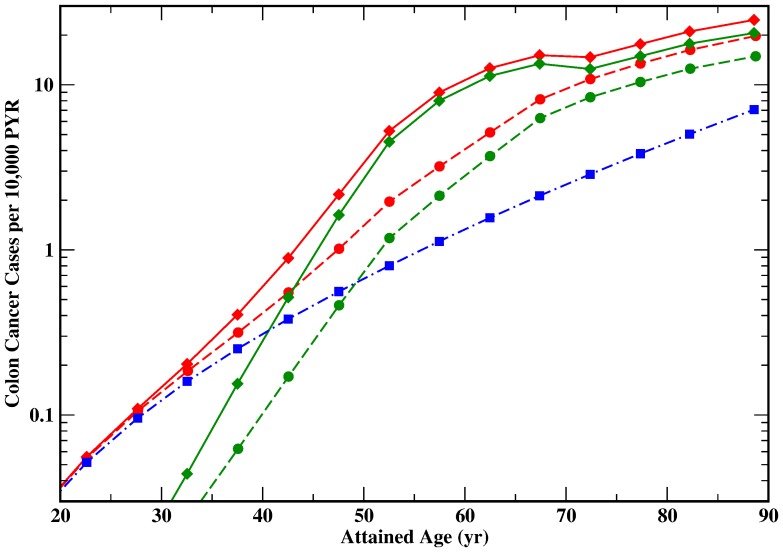
Predicted incidence rates for men (full lines) and women (dashed lines) from the two path model TP4 (red) and the contributions of the CIN pathway (green) and the MSI path (blue dot-dashed line, both sexes) in 14 intervals of attained age from 20–25 yr to 85–90 yr.

The ability to reproduce the case shares of 15–20% in the MSI pathway and 80–85% in the CIN pathway is an important test for the biological plausibility of the two path model TP4. In Table 6 the computed MSI shares are listed for the complete follow-up period and for cases recorded before and after 1980. In the early period the shares of MSI cases and CIN cases are about equal. For the later period the predicted MSI share of 17% (men 11%, women 21%) agrees remarkably well with the clinically observed data [4]. For the full period radiation generated 64 (MSI: 10) additional cases in both sexes. For women the values are 19 (MSI: 7) and for men 45 (MSI: 3). Figure 2 shows that especially for women MSI cases appear earlier than CIN cases. Also in good agreement model M3 predicted 22% MSI cases for the full period and 15% after 1980. Whereas in the CIN path models M3 and TP4 exhibit a similar radiation risk, the relative risk in the MSI path is reduced by more than a factor of two for model M3.

**Table 6:** Predicted share of cases in the MSI pathway calculated from the two path model TP4 for the full follow-up period 1958–1998 and periods 1958–1980, 1981–1998.

	Men		Women		Both sexes	
Follow-up period	Total cases	MSI share [%]	Total cases	MSI share [%]	Total cases	MSI share [%]
1958–1998	688	18	820	31	1508	25
1958–1980	161	41	206	63	367	53
1981–1998	527	11	614	21	1141	17

**Figure 3:**
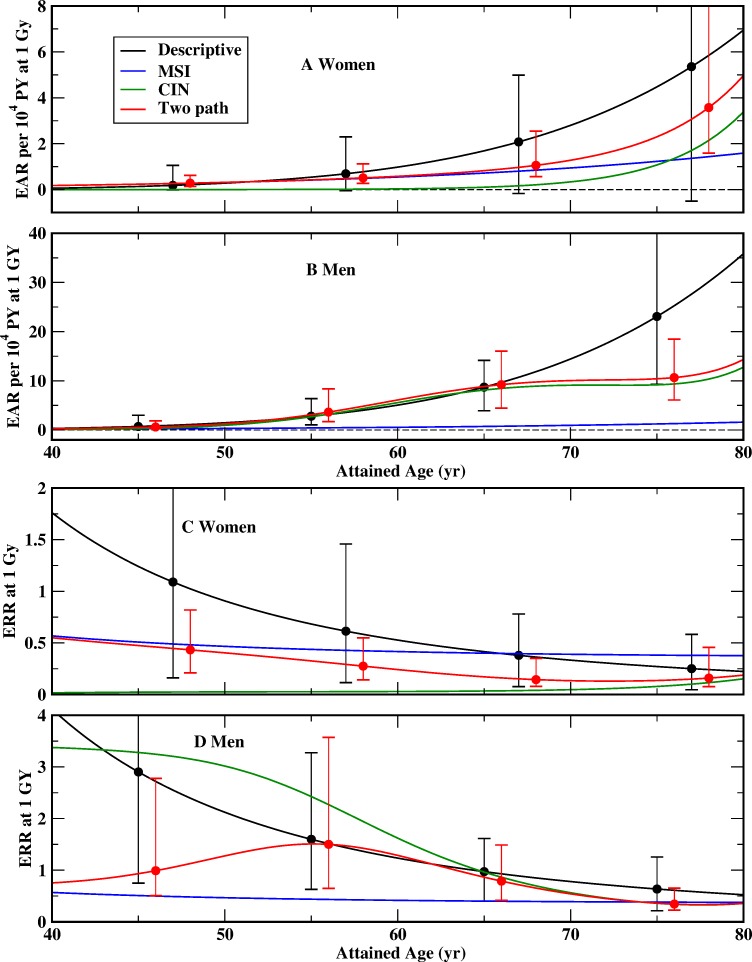
MLE with 95% CI of the excess absolute risk (EAR) per 10^4^ PY and of the excess relative risk (ERR) for women (panels A, C) and men (panels B, D), exposed to 1 Gy at age 30 (born in 1915) for the descriptive models DERR and DEAR [28] (black), and the two path model TP4 (red). Only MLE are shown for pathway-specific excess risks pertaining to MSI (blue) and CIN (green).

**Figure 4:**
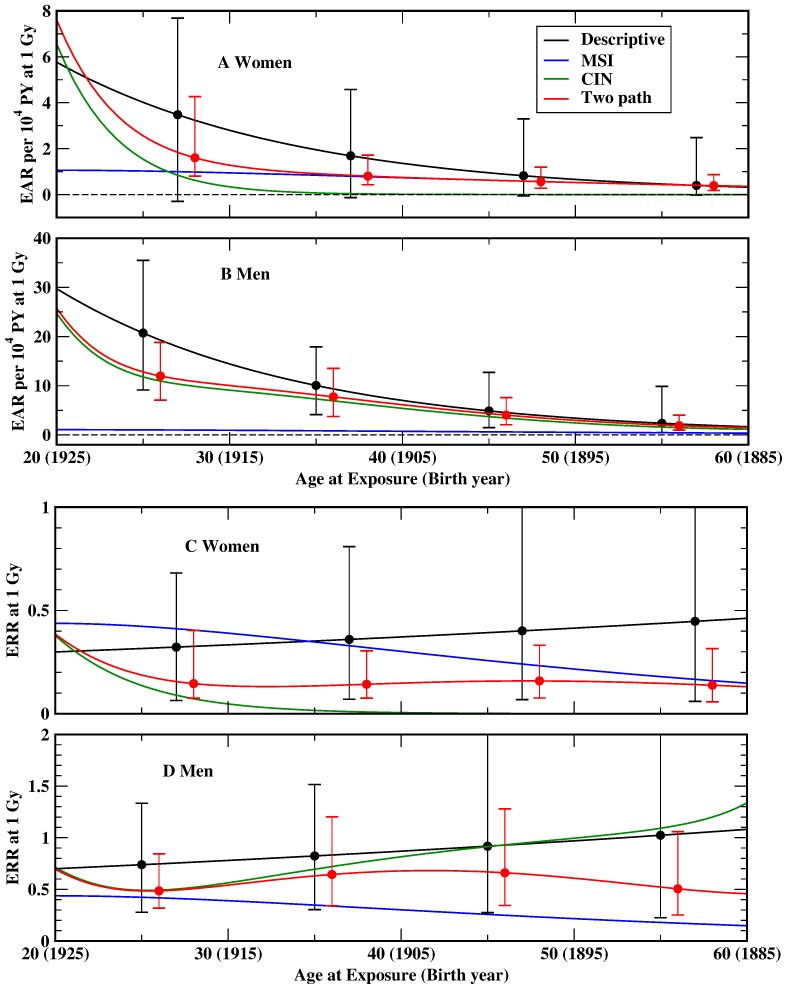
MLE with 95% CI of the excess absolute risk (EAR) per 10^4^ PY and of the excess relative risk (ERR) for women (panels A, C) and men (panels B, D) of attained age 70, exposed to 1 Gy for the descriptive models DERR and DEAR [28] (black), and the two path model TP4 (red). Only MLE are shown for pathway-specific excess risks pertaining to MSI (blue) and CIN (green).

Models DERR and DEAR are considered as the quasi-standard for radiation risk assessment. In general, the estimates for the EAR and the ERR are predicted lower by two path model TP4 compared to the descriptive models DERR and DEAR (Figures 3 and 4, Table 7). In the calculation of pathway-specific excess risks only the contribution of a single path is used.

**Table 7:** MLE (95% CI in brackets) of the excess relative risk (ERR) and the excess absolute risk (EAR) per 10^4^ PY for persons of attained age 70, exposed to 1 Gy at age 30 (born in 1915) from the descriptive models DEAR and DERR [28] and the two path model TP4.

	ERR		EAR per 10^4^ PY	
Model	Men	Women	Men	Women
Descriptive	0.77 (0.29; 1.4)	0.33 (0.066; 0.70)	14 (6.3; 24)	2.8 (−0.23; 6.4)
Two path	0.54 (0.32; 0.95)	0.13 (0.073; 0.35)	10 (5.3; 16)	1.3 (0.67; 3.2)
MSI path	0.39 (0.21; 0.68)		0.94 (0.42; 1.7)	
CIN path	0.56 (0.31; 1.1)	0.047 (0.0095; 0.27)	9.1 (4.4; 15)	0.34 (0.057; 2.2)

## Discussion

### Biological plausibility of the two path model

Loss of heterozygosity (LOH) in the *APC* gene and silencing of the *MLH1* gene evolve on a similar time scale [3]. The estimate of the initial mutation rate ν_I_ (Table 5) agrees well with a recent estimate of about 10^−5^ yr^−1^ per stem cell for the somatic mutation rate in the *APC* gene [54], but exceeds older estimates [15], [55] by an order of magnitude. Germline mutations (i.e. from *APC+/+* to *APC+/−* prior to CIN) can occur in both pathways but have not been considered explicitly in the two path model. The rate of unicryptal LOH in *MLH1* has been estimated to 2×10^−5^ yr^−1^ per stem cell from data of HNPCC patients [53]. Hence, the assumption of similar rates for early events in the MSI and CIN pathways appears justified on both biological and statistical grounds.

The estimated rate of 0.057 yr^−1^ for clonal growth in early carcinogenesis implies a rate of about one event in 18 years (or a doubling time of 12 years). Crypt fission dominates the growth dynamics in the healthy colon with a similar rate, suggesting that crypt cycle dynamics is reflected in the incidence data as the first round of clonal expansion [37]. Crypt fission is not influenced by carcinogenesis but distributes inactivated TSGs in the colon [39]. Therefore, the same rate of early clonal expansion must pertain to the MSI and CIN pathways. The biological argument is supported by a statistical criterion. Allowing different rates of early clonal expansion in both pathways did not improve the fit significantly.

The mean sojourn time from the birth of a non-extinct premalignant clone to the appearance of at least one malignant cell is given by *T_I_* = −In*(δ_I_/γ_I_^2^)/γ_I_* in the MSI pathway [19]. Inserting the MLEs of Table 4 for the identifiable parameters δ_I_ and γ_I_ yields T_I_ = 94 yr which exceeds mean human lifetime. The growth dynamics of clones in crypt fission is too slow to produce tumors of clinically relevant size. This conflict of time scales demands involvement of at least one more round of clonal expansion in cancer induction [56]. With MSCE models it has been shown that the introduction of a lag time substitutes the explicit modelling of tumor growth with a second round of clonal expansion in proper approximation [20]. The corresponding lag time has been estimated to 5–6 yr in the SEER cohort. A study of interval cancers, which develop in the time interval between serial colonoscopy, showed that MSI cancers are found four times more likely among interval cancers than among non-interval cancers [57]. These observations suggest rapid growth of malignant MSI clones after a slow development of benign adenoma. Therefore, a shorter lag time of about 3 yr should be expected in the MSI model. But models fits showed almost no dependence on lag time in the LSS cohort so that a fixed value of 5 yr was kept for both pathways.

The ratio of proliferating cells to apoptotic cells is smaller in early adenoma compared to late adenoma with high-grade displasia [58]. Other studies report an increase of both apoptosis and proliferation in colonic neoplasms which results in enhanced turnover rates [59]. These observations are compatible with higher estimates of about 0.23 yr^−1^ (women) and 0.27 yr^−1^ (men) for clonal growth in adenoma with CIN cells compared to model M3 with just one phase of clonal growth. The small sex difference in these promotion rates is highly significant, possibly due to different causes of the disease in men and women [12]. But also the altered oestrogen status after menopause might explain slower growth of adenoma which appear in women later in life [60]. A similar hormonal effect has been observed for neoplastic lesions which precede breast cancer in female a-bomb survivors [61]. Note, that the difference in a single biological model parameter fully explains the difference in the baseline incidence of men and women.

The larger number of cases in the CIN pathway allows a better resolution of the tumorigenic processes so that a second phase of clonal expansion in late adenoma could be detected. The sojourn time from the development of a small adenoma (containing CIN properties and surviving extinction) to the birth of the first malignant cell is expressed by *T_CIN_* = −In(*δ_CIN_/γ_CIN_^2^)/γ_CIN_* [20]. With the sex-specific MLEs from Table 4 sojourn times of T_CIN_ = 52 yr (men) and 58 yr (women) are calculated. In the SEER cohort a similar sojourn time (T_1_^eff^ in Table 1 of ref. [20]) of 51 yr has been found for men, but for women the value of 49 yr is lower than in the LSS cohort.

The last event before the appearance of the first malignant cells is often associated with inactivation of gene *TP53* [1]. It has been suggested that this event is very rare, so that the estimated small value of 1.5×10^−9^ yr^−1^ for the transforming mutation rate ν_CIN_ could pertain to inactivation of *TP53* (or of another gene downstream in the CIN pathway) [15].

### Lifestyle trends

In many mechanistic models of the SEER cohort the hazard functions have been adjusted descriptively for secular trends in calendar year or birth year [15], [17], [19]–[21], [62]. A statistical interpretation of this adjustment is based on the separation of secular trends from the “natural” carcinogenic mechanism. There is, however, a biological interpretation of this correction which often (depending on the mathematical model structure) amounts to assuming an impact on early TSG mutations. Since lifestyle-dependent health risks appear later in life, a biological effect on later carcinogenic events has also been tested in the present study. Firstly, an exponential dependence on birth year was applied to the initiating mutations or to the MSI mutation in the two path model. This adjustment did not improve the fit significantly (i.e. by more than 3.8 deviance points). However, the rate λ_CIN_ of the destabilizing CIN event (related to chromosomal gain or loss) grew exponentially in subsequent birth cohorts, the deviance fell by some hundred points. Rising red meat intake in a westernized diet may have increased the risk of colon cancer in Japan after the Second World War [13]. In European patients red meat intake was inversely associated with high-level MSI tumors when compared to population-based controls and positively associated with low-level MSI/CIN tumors [63]. Feeding a western diet of high fat content and low levels of calcium and vitamin D to *APC* knock-out mice resulted in a higher risk of cancer in the CIN pathway [64]. If westernized diet affected molecular sub-types similarly in Japan, it could explain the pronounced increase of CIN cases after 1980 (Table 6).

### Hallmarks of CIN in the two path model

The rate of early clonal expansion in the two path model occurs on a time scale which is reported for crypt fission [37]. This process is capable of spreading inactivated TSGs in the normal human colon and of amplifying their number [39]. If the number of cells with mutated *APC* genes increases in the CIN path, WNT signalling can no longer be suppressed effectively [65]. Up-regulation of this signalling network may precede or even cause the generation of cells carrying a CIN property. Chromosomal gain or loss compared to the normal karyotype has been identified as a prominent property of CIN cells, and the rate of gain or loss of a single chromosome has been measured to one per five stem cell divisions *in vitro* [35]. Although a comparison with *in vitro* data must be applied with caution, this measured value falls in the range of large estimates for the rate λ_CIN_ of destabilizing events in the two path model (Table 5). Other modelling studies also find a markedly enhanced “fast” third event with a four-stage model of colon carcinogenesis [15], [19]. Although the four-stage model does not consider crypt fission explicitly, the high rate of asymmetric stem cell divisions has been interpreted as the amplification of TSG −/− cells in a crypt. CIN occurs as the consequential effect of this amplification. The present results support this interpretation and add the consideration of crypt fission in the two path model as another piece to the puzzle. But they are at variance with suggestions that the CIN property is conferred early to cells with inactivated *APC* genes [16].

### Radiation risk

Estimates of the radiation response parameters r_I_ = 19 (σCI_LP_ 13; 26) yr Gy^−1^ for both sexes in initiation and r^m^_CIN_ = 5.6 (σCI_LP_ 3.8; 7.6) yr Gy^−1^ for men in clonal growth of CIN cells are close to estimates of a simpler mechanistic model for a joint set of nine cancer sites including colon [26].

Crude data of the LSS cohort (Tables 1 and 2) and estimates from standard descriptive models suggest a markedly lower radiation risk for women compared to men [28]. In the preferred two path model TP4 this fact is explained by a negligible radio-sensitivity late in the female CIN path. Independent biological evidence for this conjecture is not known to the authors of the present study. Based on statistical criteria, a radiation response on the destabilizing event in CIN for men or a unisex radiation response in the two path model cannot be ruled out (Table 3). Probably radiation affects more if not all stages of carcinogenesis but a thorough analysis of radiation targets is beyond the scope here. If models with different radiation targets describe the data almost equally well, an approach of multi-model inference, which combines all plausible models for risk estimation, might be applied [61].

Observance of pathway-specific risks has implications for risk assessment. In combination with molecular sub-type ascertainment, consideration of pathway-specific risks will improve the accuracy of expert opinion in compensation claims [32]. If the status of MSI or CIN were testable in adenoma, the application of diagnostic (or therapeutic) radiation could be optimized with a more targeted risk-benefit analysis [33].

### Limitations of the two path model

Some 200 genes are mutated in colorectal cancer [8], [66]. Eleven mutations have been counted on average per tumor but only a few mutations are common to most tumors [2]. The two path model cannot consider the effects of this complex genomic structure in detail. A one-to-one association of model parameters to mutations in specific genes such as *APC* or to bi-allelic silencing of *MLH1* should be regarded as tentative. Since a model (M3) without early clonal expansion in the CIN path yielded only a slightly inferior description of the data, the interpretation of early clonal expansion as crypt fission should also be regarded as tentative. Mutation rates of TSGs and the rates of clonal growth during crypt fission and or in premalignant adenoma should be understood as effective phenomenological parameters.

Up to now, simplifications of tumorigenic processes are inevitable as a consequence of general issues with parameter identifiability, limited statistical power and biological insight. But remarkable agreement with molecular data for a number of processes, which have been reported in the literature over the last fifteen years, emphasizes the biological plausibility of the two path model.

## Supporting Information

File S1 Contains the following files: **Table S1.** Identifiable baseline parameters in the deterministic and stochastic versions of the two path model with MSI and CIN paths (Figure 1), biological parameters N, ν_I_, α_I_ and γ_I_ are set equal in both pathways. **Table S2**. MLE, SE from a parabolic approximation around the minimum of the likelihood function, and ΔCI_LP_ from the actual likelihood profile in the standard σ range for the identifiable parameters of model M1 (Figure S1) with relation to biological parameters, superscript m,f indicates sex-dependence, radiation-response parameters r^m,f^ on dose *D* are given for lifelong radiation effect on clonal expansion, one initial mutation rate ν_I_ increases exponentially with birth year *b* = 1945.6 – *e*, the baseline version of model M1 is mathematically equivalent to the three stage model by Meza et al. [1]. **Table S3**. MLE and SE from a parabolic approximation around the minimum of the likelihood function for the identifiable parameters of model M2 (Figure S2) with relation to biological parameters, superscript m,f indicates sex-dependence, radiation-response parameters r on dose *D* given for direct radiation effect on mutation rate λ_1_ with exposure duration of 1 week, mutation rates ν_0a_, λ_0_ and ν_0b_ increase exponentially with birth year *b* = 1945.6 – *e*, ΔCI_LP_ from the actual likelihood profile in the standard σ range could not be computed by MINUIT, the baseline version of M2 is inspired by Nowak et al. [2] and derived from Little and Li [3] (their Figure 2). **Table S4**. MLE, SE from a parabolic approximation around the minimum of the likelihood function, and ΔCI_LP_ from the actual likelihood profile in the standard σ range for the identifiable parameters of model M3 (Figure S3) with relation to biological parameters, deterministic MSI model without dependence on δ_MSI_, superscript m,f indicates sex-dependence, radiation-response parameters r_I_ and r^m^_CIN_ on dose *D* are given for an exposure duration of 1 week, one initial mutation rate ν_I,CIN_ increases exponentially with birth year *b* = 1945.6 – *e* (age at exposure). **Figure S1.** Parametrisation of model M1 with lifelong radiation action (jagged bolt) on cell inactivation β (TSG: tumor suppressor gene), one initial mutation rate ν_I_ increases exponentially with birth year *b*. **Figure S2.** Parametrisation of model M2 with radiation action (jagged bolt) on mutation rate λ_1_, mutation rates ν_0a_, λ_0_ and ν_0b_ increase exponentially with birth year *b*. **Figure S3.** Parametrisation of model M3 with deterministic MSI path (no dependence on δ_MSI_) and stochastic CIN path, radiation action (jagged bolt) on second initial mutation rate ν_I_ equal in MSI and CIN paths and on cell inactivation β_CIN_ for men only, one initial mutation rate ν_I,CIN_ increases exponentially with birth year *b*.
(DOCX)

## References

[1] Markowitz SD, Bertagnolli MM (2009) Molecular origins of cancer: Molecular basis of colorectal cancer. N Engl J Med 361: 2449–2460. 20018966 10.1056/NEJMra0804588 PMC2843693

[2] Boland CR, Goel A (2010) Microsatellite instability in colorectal cancer. Gastroenterology 138: 2073–2087 e2073. 20420947 10.1053/j.gastro.2009.12.064 PMC3037515

[3] Goel A, Boland CR (2012) Epigenetics of colorectal cancer. Gastroenterology 143: 1442–1460 e1441. 23000599 10.1053/j.gastro.2012.09.032 PMC3611241

[4] Goel A, Arnold CN, Niedzwiecki D, Chang DK, Ricciardiello L, et al (2003) Characterization of sporadic colon cancer by patterns of genomic instability. Cancer Res 63: 1608–1614. 12670912

[5] Trautmann K, Terdiman JP, French AJ, Roydasgupta R, Sein N, et al (2006) Chromosomal instability in microsatellite-unstable and stable colon cancer. Clin Cancer Res 12: 6379–6385. 17085649 10.1158/1078-0432.CCR-06-1248

[6] Muleris M, Chalastanis A, Meyer N, Lae M, Dutrillaux B, et al (2008) Chromosomal instability in near-diploid colorectal cancer: a link between numbers and structure. PLoS One 3: e1632. 18286189 10.1371/journal.pone.0001632 PMC2238794

[7] Jass JR (2007) Classification of colorectal cancer based on correlation of clinical, morphological and molecular features. Histopathology 50: 113–130. 17204026 10.1111/j.1365-2559.2006.02549.x

[8] Cancer Genome Atlas N (2012) Comprehensive molecular characterization of human colon and rectal cancer. Nature 487: 330–337. 22810696 10.1038/nature11252 PMC3401966

[9] Lynch HT, de la Chapelle A (2003) Hereditary colorectal cancer. N Engl J Med 348: 919–932. 12621137 10.1056/NEJMra012242

[10] Lynch HT, Lynch JF, Lynch PM, Attard T (2008) Hereditary colorectal cancer syndromes: molecular genetics, genetic counseling, diagnosis and management. Fam Cancer 7: 27–39. 17999161 10.1007/s10689-007-9165-5

[11] Sampson JR, Jones S, Dolwani S, Cheadle JP (2005) MutYH (MYH) and colorectal cancer. Biochem Soc Trans 33: 679–683. 16042573 10.1042/BST0330679

[12] Nakaji S, Umeda T, Shimoyama T, Sugawara K, Tamura K, et al (2003) Environmental factors affect colon carcinoma and rectal carcinoma in men and women differently. Int J Colorectal Dis 18: 481–486. 12695918 10.1007/s00384-003-0485-0

[13] Takachi R, Tsubono Y, Baba K, Inoue M, Sasazuki S, et al (2011) Red meat intake may increase the risk of colon cancer in Japanese, a population with relatively low red meat consumption. Asia Pac J Clin Nutr 20: 603–612. 22094846

[14] Yiu HY, Whittemore AS, Shibata A (2004) Increasing colorectal cancer incidence rates in Japan. Int J Cancer 109: 777–781. 14999789 10.1002/ijc.20030

[15] Luebeck EG, Moolgavkar SH (2002) Multistage carcinogenesis and the incidence of colon cancer. Proceedings of the National Academy of Sciences 99: 15095–15100. 10.1073/pnas.222118199 PMC137549 12415112

[16] Nowak MA, Komarova NL, Sengupta A, Jallepalli PV, Shih Ie M, et al (2002) The role of chromosomal instability in tumor initiation. Proc Natl Acad Sci U S A 99: 16226–16231. 12446840 10.1073/pnas.202617399 PMC138593

[17] Little MP, Li G (2007) Stochastic modelling of colon cancer: Is there a role for genomic instability? Carcinogenesis 28: 479–487. 16973671 10.1093/carcin/bgl173

[18] Little MP, Vineis P, Li G (2008) A stochastic carcinogenesis model incorporating multiple types of genomic instability fitted to colon cancer data. Journal of Theoretical Biology 254: 229–238. 18640693 10.1016/j.jtbi.2008.05.027

[19] Meza R, Jeon J, Moolgavkar SH, Luebeck EG (2008) Age-specific incidence of cancer: Phases, transitions, and biological implications. Proc Natl Acad Sci U S A 105: 16284–16289. 18936480 10.1073/pnas.0801151105 PMC2570975

[20] Luebeck EG, Curtius K, Jeon J, Hazelton WD (2013) Impact of tumor progression on cancer incidence curves. Cancer Res 73: 1086–1096. 23054397 10.1158/0008-5472.CAN-12-2198 PMC3746830

[21] Meza R, Jeon J, Renehan AG, Luebeck EG (2010) Colorectal cancer incidence trends in the United States and United kingdom: evidence of right- to left-sided biological gradients with implications for screening. Cancer Res 70: 5419–5429. 20530677 10.1158/0008-5472.CAN-09-4417 PMC2914859

[22] Morgan WF (2003) Non-targeted and delayed effects of exposure to ionizing radiation: I. Radiation-induced genomic instability and bystander effects in vitro. Radiat Res 159: 567–580. 12710868 10.1667/0033-7587(2003)159[0567:nadeoe]2.0.co;2

[23] Morgan WF (2003) Non-targeted and delayed effects of exposure to ionizing radiation: II. Radiation-induced genomic instability and bystander effects in vivo, clastogenic factors and transgenerational effects. Radiat Res 159: 581–596. 12710869 10.1667/0033-7587(2003)159[0581:nadeoe]2.0.co;2

[24] Kadhim M, Salomaa S, Wright E, Hildebrandt G, Belyakov OV, et al (2013) Non-targeted effects of ionising radiation-Implications for low dose risk. Mutat Res 752: 84–98. 23262375 10.1016/j.mrrev.2012.12.001 PMC4091999

[25] Jacob P, Meckbach R, Kaiser JC, Sokolnikov M (2010) Possible expressions of radiation-induced genomic instability, bystander effects or low-dose hypersensitivity in cancer epidemiology. Mutat Res 687: 34–39. 20096708 10.1016/j.mrfmmm.2010.01.005

[26] Heidenreich WF, Cullings HM, Funamoto S, Paretzke HG (2007) Promoting action of radiation in the atomic bomb survivor carcinogenesis data? Radiat Res 168: 750–756. 18088179 10.1667/RR0919.1

[27] Shuryak I, Sachs RK, Brenner DJ (2010) Cancer risks after radiation exposure in middle age. J Natl Cancer Inst 102: 1628–1636. 20975037 10.1093/jnci/djq346 PMC2970575

[28] Preston DL, Ron E, Tokuoka S, Funamoto S, Nishi N, et al (2007) Solid cancer incidence in atomic bomb survivors: 1958–1998. Radiat Res 168: 1–64. 17722996 10.1667/RR0763.1

[29] BEIR (2006) Health risks from exposure to low levels of ionizing radiation: BEIR VII - phase 2. Washington, D1 C.: United States National Academy of Sciences, National Academy Press.

[30] ICRP (2007) The 2007 Recommendations of the International Commission on Radiological Protection; Valentin J, editor: Elsevier.

[31] UNSCEAR (2008) 2006 Report I, Effects of Ionizing Radiation. New York: United Nations.

[32] Kocher DC, Apostoaei AI, Henshaw RW, Hoffman FO, Schubauer-Berigan MK, et al (2008) Interactive RadioEpidemiological Program (IREP): a web-based tool for estimating probability of causation/assigned share of radiogenic cancers. Health Phys 95: 119–147. 18545036 10.1097/01.HP.0000291191.49583.f7 PMC4018571

[33] Berrington de Gonzalez A, Kim KP, Knudsen AB, Lansdorp-Vogelaar I, Rutter CM, et al (2011) Radiation-related cancer risks from CT colonography screening: a risk-benefit analysis. AJR Am J Roentgenol 196: 816–823. 21427330 10.2214/AJR.10.4907 PMC3470483

[34] Cullings HM, Fujita S, Funamoto S, Grant EJ, Kerr GD, et al (2006) Dose estimation for atomic bomb survivor studies: its evolution and present status. Radiat Res 166: 219–254. 16808610 10.1667/RR3546.1

[35] Lengauer C, Kinzler KW, Vogelstein B (1997) Genetic instabilities in colorectal cancers. Nature 386: 623–627. 9121588 10.1038/386623a0

[36] Kinzler KW, Vogelstein B (1997) Cancer-susceptibility genes. Gatekeepers and caretakers. Nature 386: 761–763. 9126728 10.1038/386761a0

[37] Humphries A, Wright NA (2008) Colonic crypt organization and tumorigenesis. Nat Rev Cancer 8: 415–424. 18480839 10.1038/nrc2392

[38] Loeffler M, Bratke T, Paulus U, Li YQ, Potten CS (1997) Clonality and life cycles of intestinal crypts explained by a state dependent stochastic model of epithelial stem cell organization. J Theor Biol 186: 41–54. 9176636 10.1006/jtbi.1996.0340

[39] Greaves LC, Preston SL, Tadrous PJ, Taylor RW, Barron MJ, et al (2006) Mitochondrial DNA mutations are established in human colonic stem cells, and mutated clones expand by crypt fission. Proc Natl Acad Sci U S A 103: 714–719. 16407113 10.1073/pnas.0505903103 PMC1325106

[40] Heidenreich WF, Paretzke HG (2008) Promotion of initiated cells by radiation-induced cell inactivation. Radiat Res 170: 613–617. 18959457 10.1667/RR0957.1

[41] Akaike H (1973) Information theory and extension of the maximum likelihood principle. Proceedings of the Second International Symposium on Information Theory. Budapest, Hungary: Akademiai Kiado. pp. 267–281.

[42] Little MP, Wright EG (2003) A stochastic carcinogenesis model incorporating genomic instability fitted to colon cancer data. Math Biosci 183: 111–134. 12711407 10.1016/s0025-5564(03)00040-3

[43] Moolgavkar SH, Knudson AG Jr (1981) Mutation and cancer: a model for human carcinogenesis. J Natl Cancer Inst 66: 1037–1052. 6941039 10.1093/jnci/66.6.1037

[44] Feller W (1968) An Introduction to Probability Theory and Its Applications. New York: Wiley.

[45] Heidenreich WF, Luebeck EG, Moolgavkar SH (1997) Some properties of the hazard function of the two-mutation clonal expansion model. Risk Analysis 17: 391–399. 9232020 10.1111/j.1539-6924.1997.tb00878.x

[46] Little MP, Heidenreich WF, Li G (2010) Parameter identifiability and redundancy: theoretical considerations. PLoS One 5: e8915. 20111720 10.1371/journal.pone.0008915 PMC2811744

[47] Kaiser JC (2010) MECAN - A Software Package to Estimate Health Risks in Radiation Epidemiology with Multi-Model Inference, User's Guide. Neuherberg, Germany.

[48] James F (1994) MINUIT - Function minimization and error analysis, version 94.1, CERN Program Library Entry D506. Geneva

[49] Preston DL, Lubin JH, Pierce DA (1993) Epicure User's Guide. Seattle (WA)..

[50] Kaiser JC, Walsh L (2013) Independent analysis of the radiation risk for leukaemia in children and adults with mortality data (1950–2003) of Japanese A-bomb survivors. Radiat Environ Biophys 52: 17–27. 23124826 10.1007/s00411-012-0437-6 PMC3579470

[51] Potten CS, Booth C, Hargreaves D (2003) The small intestine as a model for evaluating adult tissue stem cell drug targets. Cell Proliferation 36: 115–129. 12814429 10.1046/j.1365-2184.2003.00264.x PMC6496932

[52] Moolgavkar SH, Luebeck EG (1992) Multistage carcinogenesis: population-based model for colon cancer. J Natl Cancer Inst 84: 610–618. 1313509 10.1093/jnci/84.8.610

[53] Herrero-Jimenez P, Tomita-Mitchell A, Furth EE, Morgenthaler S, Thilly WG (2000) Population risk and physiological rate parameters for colon cancer. The union of an explicit model for carcinogenesis with the public health records of the United States. Mutat Res 447: 73–116. 10686307 10.1016/s0027-5107(99)00201-8

[54] Hornsby C, Page KM, Tomlinson I (2008) The in vivo rate of somatic adenomatous polyposis coli mutation. Am J Pathol 172: 1062–1068. 18321998 10.2353/ajpath.2008.070724 PMC2276411

[55] Iwama T (2001) Somatic mutation rate of the APC gene. Jpn J Clin Oncol 31: 185–187. 11450991 10.1093/jjco/hye042

[56] Jones S, Chen WD, Parmigiani G, Diehl F, Beerenwinkel N, et al (2008) Comparative lesion sequencing provides insights into tumor evolution. Proc Natl Acad Sci U S A 105: 4283–4288. 18337506 10.1073/pnas.0712345105 PMC2393770

[57] Sawhney MS, Farrar WD, Gudiseva S, Nelson DB, Lederle FA, et al (2006) Microsatellite instability in interval colon cancers. Gastroenterology 131: 1700–1705. 17087932 10.1053/j.gastro.2006.10.022

[58] Koike M (1996) Significance of spontaneous apoptosis during colorectal tumorigenesis. J Surg Oncol 62: 97–108. 8649048 10.1002/(SICI)1096-9098(199606)62:2<97::AID-JSO5>3.0.CO;2-L

[59] Koornstra JJ, de Jong S, Hollema H, de Vries EG, Kleibeuker JH (2003) Changes in apoptosis during the development of colorectal cancer: a systematic review of the literature. Crit Rev Oncol Hematol 45: 37–53. 12482571 10.1016/s1040-8428(01)00228-1

[60] Foster PA (2013) Oestrogen and colorectal cancer: mechanisms and controversies. Int J Colorectal Dis 28: 737–749. 23319136 10.1007/s00384-012-1628-y

[61] Kaiser JC, Jacob P, Meckbach R, Cullings HM (2012) Breast cancer risk in atomic bomb survivors from multi-model inference with incidence data 1958–1998. Radiat Environ Biophys 51: 1–14. 21947564 10.1007/s00411-011-0387-4

[62] Little MP (2008) Leukaemia Following Childhood Radiation Exposure In The Japanese Atomic Bomb Survivors And In Medically Exposed Groups. Radiation Protection Dosimetry 132: 156–165. 18936088 10.1093/rpd/ncn264

[63] Diergaarde B, Braam H, van Muijen GNP, Ligtenberg MJL, Kok FJ, et al (2003) Dietary Factors and Microsatellite Instability in Sporadic Colon Carcinomas. Cancer Epidemiology, Biomarkers and Prevention 12: 1130–1136. 14652271

[64] Yang K, Edelmann W, Fan K, Lau K, Leung D, et al (1998) Dietary Modulation of Carcinoma Development in a Mouse Model for Human Familial Adenomatous Polyposis. Cancer Research 58: 5713–5717. 9865728

[65] Boman BM, Fields JZ (2013) An APC:WNT Counter-Current-Like Mechanism Regulates Cell Division Along the Human Colonic Crypt Axis: A Mechanism That Explains How Mutations Induce Proliferative Abnormalities That Drive Colon Cancer Development. Front Oncol 3: 244. 24224156 10.3389/fonc.2013.00244 PMC3819610

[66] Sjoblom T, Jones S, Wood LD, Parsons DW, Lin J, et al (2006) The consensus coding sequences of human breast and colorectal cancers. Science 314: 268–274. 16959974 10.1126/science.1133427

