# Supplementary material for: Genomic Instability and Radiation Risk in Molecular Pathways to Colon Cancer
Source: PLoS One. 2014 Oct 30;9(10):e111024. doi: 10.1371/journal.pone.0111024 (PMC4214691; doi:10.1371/journal.pone.0111024)
Supplement: File S1 — Contains the following files: Table S1. Identifiable baseline parameters in the deterministic and stochastic versions of the two path model with MSI and CIN paths (Figure 1), biological parameters N, νI, αI and γI are set equal in both pathways. Table S2. MLE, SE from a parabolic approximation around the minimum of the likelihood function, and ΔCILP from the actual likelihood profile in the standard σ range for the identifiable parameters of model M1 (Figure S1) with relation to biological parameters, superscript m,f indicates sex-dependence, radiation-response parameters rm,f on dose D are given for lifelong radiation effect on clonal expansion, one initial mutation rate νI increases exponentially with birth year b = 1945.6 – e, the baseline version of model M1 is mathematically equivalent to the three stage model by Meza et al. [1]. Table S3. MLE and SE from a parabolic approximation around the minimum of the likelihood function for the identifiable parameters of model M2 (Figure S2) with relation to biological parameters, superscript m,f indicates sex-dependence, radiation-response parameters r on dose D given for direct radiation effect on mutation rate λ1 with exposure duration of 1 week, mutation rates ν0a, λ0 and ν0b increase exponentially with birth year b = 1945.6 – e, ΔCILP from the actual likelihood profile in the standard σ range could not be computed by MINUIT, the baseline version of M2 is inspired by Nowak et al. [2] and derived from Little and Li [3] (their Figure 2). Table S4. MLE, SE from a parabolic approximation around the minimum of the likelihood function, and ΔCILP from the actual likelihood profile in the standard σ range for the identifiable parameters of model M3 (Figure S3) with relation to biological parameters, deterministic MSI model without dependence on δMSI, superscript m,f indicates sex-dependence, radiation-response parameters rI and rm CIN on dose D are given for an exposure duration of 1 week, one initial mutation rate νI,CIN inc [file pone.0111024.s001.docx]

**Supplementary Information:
Genetic instability and radiation risk in molecular pathways to colon cancer
by J.C. Kaiser, R. Meckbach and P. Jacob
PONE-D-14-042321**

**Table S1.** Identifiable baseline parameters in the deterministic and stochastic versions of the two path model with MSI and CIN paths (Figure 1), biological parameters N, ν_I_, α_I_ and γ_I_ are set equal in both pathways.

| **Symbol** | **Unit** | **Relation to biological parameters** | **Remarks** |
| --- | --- | --- | --- |
| **Deterministic model for MSI path** | | | |
| R_MSI_ | yr^-3^ | = Nν_I_^2^ν_MSI_ |  |
| γ_I_ | yr^-1^ | = α_I_ – β_I_ | equal in MSI and CIN |
| **Deterministic model for CIN path** | | | |
| R_MSI_ | yr^-4^ | = Nν_I_^2^λ_CIN_ν_CIN_ |  |
| γ_I_ | yr^-1^ | = α_I_ – β_I_ | equal in MSI and CIN |
| γ_CIN_ | yr^-1^ | = α_CIN_ – β_CIN_ |  |
| **Stochastic model for MSI path** | | | |
| R_MSI_ | yr^-3^ | = Nν_I_^2^ν_MSI_ |  |
| γ_I_ | yr^-1^ | = α_I_ – β_I_ – ν_MSI_ | equal in MSI and CIN |
| δ_I_ | yr^-2^ | = α_I_ ν_MSI_ |  |
| ρ_MSI_ | yr^-2^ | = ν_I_ν_MSI_ | practically not identifiable |
| **Stochastic model for CIN path** | | | |
| R_CIN_ | yr^-3^ | = Nν_I_^2^ λ_CIN_ν_CIN_ |  |
| γ_I_ | yr^-1^ | = α_I_ – β_I_ | equal in MSI and CIN for γ_I_ >> ν_MSI_ |
| δ*_I_ | yr^-3^ | = α_I_ λ_CIN_ν_CIN_ | determined by R_CIN_/ R_MSI_ δ_I_ |
| γ_CIN_ | yr^-1^ | = α_CIN_ – β_CIN_ – ν_CIN_ |  |
| δ_CIN_ | yr^-2^ | = α_CIN_ ν_CIN_ |  |
| ρ_1_ | yr^-3^ | = ν_I_ λ_CIN_ν_CIN_ | practically not identifiable |
| ρ_CIN_ | yr^-2^ | = λ_CIN_ν_CIN_ | practically not identifiable |

**Table S2**. MLE, SE from a parabolic approximation around the minimum of the likelihood function, and ΔCI_LP_ from the actual likelihood profile in the standard σ range for the identifiable parameters of model M1 (Figure S1) with relation to biological parameters, superscript m,f indicates sex-dependence, radiation-response parameters r^m,f^ on dose *D* are given for lifelong radiation effect on clonal expansion, one initial mutation rate ν_I_ increases exponentially with birth year *b* = 1945.6 – *e,* the baseline version of model M1 is mathematically equivalent to the three stage model by Meza et al. [1].

| **Symbol** | **Unit** | **MLE** | **SE** | **ΔCI_LP_** | **Relation to biological parameters** |
| --- | --- | --- | --- | --- | --- |
| R_0_ | yr^-3^ | -21.39^a^ | 0.37 | -0.34; 0.32 | = Nν_I_^2^ν_T_ f(1916.5) |
| l_b_ | yr^-1^ | -0.0627 | 0.0027 | -0.0027, 0.0027 | f(*b*) = exp[l_b_ (1915.6 - *b*)] |
| γ^m^_0_ | yr^-1^ | 0.1770 | 0.0085 | -0.0074; 0.0078 | = α – β^m^ g^m^(0) – ν_T_ |
| γ^f^_0_ | yr^-1^ | 0.1500 | 0.0078 | -0.0068; 0.0072 | = α – β^f^ g^f^(0) – ν_T_ |
| δ | yr^-2^ | -14.91^a^ | 0.28 | -0.27; 0.26 | = α ν_T_ |
| r^m^ | Gy^-1^ | 0.143 | 0.025 | -0.025; 0.026 | g^m^(*D*) = 1-r^m^ *D* |
| r^f^ | Gy^-1^ | 0.052 | 0.029 | -0.030; 0.029 | g^f^(*D*) = 1-r^f^ *D* |

^a^log-transformed

**Table S3**. MLE and SE from a parabolic approximation around the minimum of the likelihood function for the identifiable parameters of model M2 (Figure S2) with relation to biological parameters, superscript m,f indicates sex-dependence, radiation-response parameters r on dose *D* given for direct radiation effect on mutation rate λ_1_ with exposure duration of 1 week, mutation rates ν_0a_, λ_0_ and ν_0b_ increase exponentially with birth year *b* = 1945.6 – *e,* ΔCI_LP_ from the actual likelihood profile in the standard σ range could not be computed by MINUIT, the baseline version of M2 is inspired by Nowak et al. [2] and derived from Little and Li [3] (their Figure 2).

| **Symbol** | **Unit** | **MLE** | **SE** | **Relation to biological parameters** |
| --- | --- | --- | --- | --- |
| R^m^_ab0_ | yr^-3^ | -26.12^a^ | 0.44 | = Nν^m^_0a_λ^m^_1_(0) ν_1b_ f(1915.6) |
| R^f^_ab0_ | yr^-3^ | -29.82^a^ | 0.54 | = Nν^f^_0a_λ^f^_1_(0) ν_1b_ f(1915.6) |
| R^m^_b0_ | yr^-3^ | -22.09^a^ | 0.41 | = Nλ_0_ν^m^_0b_ ν_1b_ f^2^(1915.6) |
| R^f^_b0_ | yr^-3^ | -20.79^a^ | 0.32 | = Nλ_0_ν^f^_0b_ ν_1b_ f^2^(1915.6) |
| l_b_ | yr^-1^ | -0.0332 | 0.0014 | f(*b*) = exp[l_b_ (1915.6 - *b*)] |
| γ^m^_1a_ | yr^-1^ | 0.162 | 0.019 | = α_1a_ – β^m^_1a_ |
| γ^f^_1a_ | yr^-1^ | 0.340 | 0.024 | = α_1a_ – β^f^_1a_ |
| δ_1a_ | yr^-3^ | -22.4^a^ | fixed | = α_1a_ λ_1,0_ ν_1b_ |
| γ^m^_1b_ | yr^-1^ | 0.217 | 0.013 | = α_1b_ – β^m^_1b_ – ν_1b_ |
| γ^f^_1b_ | yr^-1^ | 0.156 | 0.009 | = α_1b_ – β^f^_1b_ – ν_1b_ |
| δ_1b_ | yr^-2^ | -14.65^a^ | fixed | = α_1b_ ν_1b_ |
| r^m^ | week Gy^-1^ | 10.65^a^ | 0.84 | λ^m^_1_(*D*) = λ_1,0_ (1+r^m^ *D/*week) |
| r^f^ | week Gy^-1^ | 14.79^a^ | 1.23 | λ^f^_1_(*D*) = λ_1,0_ (1+r^f^ *D/*week) |
| t_lag_ | Yr | 5 | fixed |  |

^a^log-transformed

**Table S4**. MLE, SE from a parabolic approximation around the minimum of the likelihood function, and ΔCI_LP_ from the actual likelihood profile in the standard σ range for the identifiable parameters of model M3 (Figure S3) with relation to biological parameters, deterministic MSI model without dependence on δ_MSI_, superscript m,f indicates sex-dependence, radiation-response parameters r_I_ and r^m^_CIN_ on dose *D* are given for an exposure duration of 1 week, one initial mutation rate ν_I,CIN_ increases exponentially with birth year *b* = 1945.6 – *e* (age at exposure).

| **Symbol** | **Unit** | **MLE** | **SE** | **ΔCI_LP_** | **Relation to biological parameters** |
| --- | --- | --- | --- | --- | --- |
| **Deterministic model for MSI path** | | | | | |
| R_MSI_ | yr^-3^ | -18.27^a^ | 0.77 | -0.58; 0.44 | = N_MSI_ν_I,MSI_^2^ν_T,MSI_ r(0) |
| γ_MSI_ | yr^-1^ | 0.069 | 0.014 | -0.011, 0.011 | = α_MSI_ – β_MSI_ |
| **Stochastic model for CIN path** | | | | | |
| R_CIN_ | yr^-3^ | -22.24^a^ | 1.21 | -0.65; 0.60 | = N_CIN_ν_I,CIN_^2^ν_T,CIN_ r(0) f(1915.6) |
| l_b_ | yr^-1^ | -0.0835 | 0.0086 | -0.0067; 0.0064 | f(*b*) = exp[l_b_ (1915.6 - *b*)] |
| γ^m^_CIN_ | yr^-1^ | 0.208 | 0.025 | -0.013; 0.014 | = α_CIN_ – β^m^_CIN_ g(0) – ν_T,CIN_ |
| γ^f^_CIN_ | yr^-1^ | 0.186 | 0.023 | -0.011; 0.013 | = α_CIN_ – β^f^_CIN_ – ν_T,CIN_ |
| δ_CIN_ | yr^-2^ | -15.43^a^ | 0.98 | -0.55; 0.52 | = α_CIN_ ν_T,CIN_ |
| r^m^_CIN_ | week Gy^-1^ | 5.44^a^ | 0.36 | -0.43; 0.31 | g(*D*) = 1-r^m^_CIN_ *D*/week |
| **MSI and CIN paths** | | | | | |
| r_I_ | week Gy^-1^ | 6.21^a^ | 0.70 | -0.87; 0.57 | r(*D*) = 1+ r_I_ *D*/week |
| t_lag_ | yr | 5 (fixed) |  |  |  |

^a^log-transformed

**Figure S1.** Parametrisation of model M1 with lifelong radiation action (jagged bolt) on cell inactivation β (TSG: tumor suppressor gene), one initial mutation rate ν_I_ increases exponentially with birth year *b*.

**Figure S2.** Parametrisation of model M2 with radiation action (jagged bolt) on mutation rate λ_1_, mutation rates ν_0a_, λ_0_ and ν_0b_ increase exponentially with birth year *b*.

**Figure S3.** Parametrisation of model M3 with deterministic MSI path (no dependence on δ_MSI_) and stochastic CIN path, radiation action (jagged bolt) on second initial mutation rate ν_I_ equal in MSI and CIN paths and on cell inactivation β_CIN_ for men only, one initial mutation rate ν_I,CIN_ increases exponentially with birth year *b*.

**References**

1. Meza R, Jeon J, Moolgavkar SH, Luebeck EG (2008) Age-specific incidence of cancer: Phases, transitions, and biological implications. Proc Natl Acad Sci U S A 105: 16284-16289.

2. Nowak MA, Komarova NL, Sengupta A, Jallepalli PV, Shih Ie M, et al. (2002) The role of chromosomal instability in tumor initiation. Proc Natl Acad Sci U S A 99: 16226-16231.

3. Little MP, Li G (2007) Stochastic modelling of colon cancer: Is there a role for genomic instability? Carcinogenesis 28: 479-487.
